# Supplementary material for: MetaRibo-Seq measures translation in microbiomes
Source: Nat Commun. 2020 Jun 29;11:3268. doi: 10.1038/s41467-020-17081-z (PMC7324362; doi:10.1038/s41467-020-17081-z)
Supplement: Supplementary file 10 — Supplementary Data 7 [file 41467_2020_17081_MOESM10_ESM.zip › File2/Confidence_High_Taxonomy/437985_out.krona.html]

Javascript must be enabled to view this page.

members
magnitude
magnitudeUnassigned
count
unassigned
taxon
rank

437985\_out

122

2
superkingdom
122

122
phylum
976

200643
class
122

171549
order
122

1
family
815

genus
1
816


SRS142890\_contig\_number\_24302
2292944
1
species

121
family
1853231


SRS013521\_contig\_number\_10637SRS015264\_contig\_number\_7007SRS019381\_contig\_number\_19793SRS020394\_contig\_number\_contig-100\_100.85282SRS048060\_contig\_number\_3306SRS056259\_contig\_number\_4431SRS077231\_contig\_number\_19654SRS077641\_contig\_number\_contig-100\_545.121776SRS143991\_contig\_number\_5874SRS893341\_contig\_number\_12995
genus
10
283168
121


SRS016018\_contig\_number\_6303SRS043768\_contig\_number\_27745SRS046068\_contig\_number\_2317SRS050026\_contig\_number\_contig-100\_354.73011SRS058770\_contig\_number\_contig-100\_117.153837SRS062654\_contig\_number\_850SRS064276\_contig\_number\_35995SRS077502\_contig\_number\_contig-100\_1585.86837SRS077849\_contig\_number\_contig-100\_269.186038SRS078665\_contig\_number\_237SRS097958\_contig\_number\_contig-100\_207.151818SRS147139\_contig\_number\_37961
1637502
12
species

1263090

SRS077086\_contig\_number\_8051
species
1


SRS011084\_contig\_number\_15010SRS011134\_contig\_number\_27144SRS011239\_contig\_number\_1SRS012849\_contig\_number\_5SRS012969\_contig\_number\_1SRS013098\_contig\_number\_451SRS013476\_contig\_number\_35265SRS013800\_contig\_number\_21118SRS013965\_contig\_number\_22314SRS014287\_contig\_number\_10064SRS014683\_contig\_number\_138SRS015431\_contig\_number\_23SRS015663\_contig\_number\_5SRS015890\_contig\_number\_65SRS016056\_contig\_number\_3898SRS016095\_contig\_number\_213SRS016954\_contig\_number\_1856SRS017247\_contig\_number\_4844SRS017433\_contig\_number\_2938SRS017821\_contig\_number\_494SRS018133\_contig\_number\_11813SRS018817\_contig\_number\_3SRS018836\_contig\_number\_13970SRS018888\_contig\_number\_10016SRS018984\_contig\_number\_54SRS019030\_contig\_number\_101SRS019068\_contig\_number\_1512SRS019161\_contig\_number\_3SRS019397\_contig\_number\_106SRS020233\_contig\_number\_13003SRS020328\_contig\_number\_28465SRS021219\_contig\_number\_130SRS021484\_contig\_number\_17609SRS022609\_contig\_number\_2SRS023526\_contig\_number\_99SRS024075\_contig\_number\_10291SRS024132\_contig\_number\_51153SRS024435\_contig\_number\_57SRS043001\_contig\_number\_1069SRS043701\_contig\_number\_19SRS043841\_contig\_number\_9267SRS044535\_contig\_number\_83SRS045244\_contig\_number\_contig-100\_1988.54962SRS046369\_contig\_number\_4SRS047014\_contig\_number\_15SRS047433\_contig\_number\_14545SRS049446\_contig\_number\_13157SRS049712\_contig\_number\_10575SRS049773\_contig\_number\_232SRS049900\_contig\_number\_3250SRS050925\_contig\_number\_40SRS051031\_contig\_number\_19SRS053214\_contig\_number\_1462SRS053398\_contig\_number\_2294SRS063190\_contig\_number\_9879SRS063370\_contig\_number\_5973SRS064557\_contig\_number\_68SRS064757\_contig\_number\_63SRS065504\_contig\_number\_36103SRS075773\_contig\_number\_contig-100\_154.194418SRS076804\_contig\_number\_55SRS077127\_contig\_number\_124SRS077194\_contig\_number\_19876SRS077294\_contig\_number\_12704SRS077392\_contig\_number\_40477SRS077552\_contig\_number\_14SRS077730\_contig\_number\_5SRS078242\_contig\_number\_2196SRS078419\_contig\_number\_38SRS098571\_contig\_number\_19SRS098644\_contig\_number\_498SRS098827\_contig\_number\_59SRS098881\_contig\_number\_4SRS100021\_contig\_number\_132SRS103987\_contig\_number\_contig-100\_224.198622SRS104311\_contig\_number\_18SRS104636\_contig\_number\_23931SRS105153\_contig\_number\_2SRS143417\_contig\_number\_7SRS143598\_contig\_number\_493SRS143876\_contig\_number\_1738SRS144506\_contig\_number\_42218SRS146764\_contig\_number\_9783SRS147271\_contig\_number\_13433SRS147557\_contig\_number\_contig-100\_1826.172378SRS148091\_contig\_number\_238SRS148196\_contig\_number\_20896SRS148721\_contig\_number\_42436SRS149181\_contig\_number\_24577SRS893252\_contig\_number\_7347SRS893378\_contig\_number\_4304
28118
species
91

species
7

SRS015782\_contig\_number\_3SRS016335\_contig\_number\_11617SRS017191\_contig\_number\_8655SRS049995\_contig\_number\_2SRS058770\_contig\_number\_49SRS078176\_contig\_number\_9SRS144183\_contig\_number\_33778
1897010
